# Supramolecular structures of Ni^II^ and Cu^II^ with the sterically demanding Schiff base dyes driven by cooperative action of preagostic and other non-covalent interactions

**DOI:** 10.1107/S2052252521000610

**Published:** 2021-05-01

**Authors:** Alexey A. Shiryaev, Tatyana M. Burkhanova, Mariusz P. Mitoraj, Mercedes Kukulka, Filip Sagan, Ghodrat Mahmoudi, Maria G. Babashkina, Michael Bolte, Damir A. Safin

**Affiliations:** a University of Tyumen, Volodarskogo Street 6, Tyumen, 625003, Russian Federation; b Innovation Center for Chemical and Pharmaceutical Technologies, Ural Federal University named after the First President of Russia B. N. Yeltsin, Mira Street 19, Ekaterinburg, 620002, Russian Federation; c Kurgan State University, Sovetskaya Street 63/4, 640020, Russian Federation; d Department of Theoretical Chemistry, Faculty of Chemistry, Jagiellonian University, Gronostajowa 2, Kraków, 30-387, Poland; e Department of Chemistry, Faculty of Science, University of Maragheh, PO Box 55181-83111, Maragheh, Iran; f Institute of Condensed Matter and Nanosciences, Université Catholique de Louvain, Place L. Pasteur 1, Louvain-la-Neuve, 1348, Belgium; g Institut für Anorganische Chemie, J.-W.-Goethe-Universität, Frankfurt/Main, Germany

**Keywords:** Schiff bases, *N*-salicyl­idene aniline derivatives, UV–Vis spectroscopy, luminescence, Hirshfeld surface analysis, discrete mononuclear homoleptic complexes, preagostic interactions, non-covalent interactions

## Abstract

This work reports on synthesis and extensive experimental and theoretical investigations on photophysical, structural and thermal properties of the Ni^II^ and Cu^II^ discrete mononuclear homoleptic complexes [Ni(*L* ^I,II^)_2_] and [Cu(*L* ^I,II^)_2_] fabricated from the Schiff base dyes *o*-HOC_6_H_4_—CH=N—*cyclo*-C_6_H_11_ (H*L* ^I^) and *o*-HOC_10_H_6_—CH=N—*cyclo*-C_6_H_11_ (H*L* ^II^), containing the sterically crowding cyclo­hexyl units. The six-membered metallocycles adopt a clearly defined envelope conformation in [Ni(*L* ^II^)_2_], while they are much more planar in the structures of [Ni(*L* ^I^)_2_] and [Cu(*L* ^I,II^)_2_]. It has been demonstrated by in-depth bonding analyses based on the ETS-NOCV and Interacting Quantum Atoms energy-decomposition schemes that application of the bulky substituents, containing several C—H groups, has led to the formation of a set of classical and unintuitive intra- and inter-molecular interactions. All together they are responsible for the high stability of [Ni(*L* ^I,II^)_2_] and [Cu(*L* ^I,II^)_2_]. More specifically, London dispersion dominated intramolecular C—H⋯O, C—H⋯N and C—H⋯H—C hydrogen bonds are recognized and, importantly, the attractive, chiefly the Coulomb driven, preagostic (not repulsive anagostic) C—H⋯Ni/Cu interactions have been discovered despite their relatively long distances (∼2.8–3.1 Å). All the complexes are further stabilized by the extremely efficient intermolecular C—H⋯π(benzene) and C—H⋯π(chelate) interactions, where both the charge-delocalization and London dispersion constituents appear to be crucial for the crystal packing of the obtained complexes. All the complexes were found to be photoluminescent in CH_2_Cl_2_, with [Cu(*L* ^II^)_2_] exhibiting the most pronounced emission – the time-dependent density-functional-theory computations revealed that it is mostly caused by metal-to-ligand charge-transfer transitions.

## Abstract 2

This work shows the importance of counterintuitive ultra long distances, intramolecular preagostic attractive C—H⋯Ni/Cu and C—H⋯O, C—H⋯H—C contacts and a bunch of other extremely strong non-classic mainly London dispersion driven intermolecular interactions involving C—H bonds: C—H⋯*X* (*X* = H—C, N, O, π) in the crystal structures of the Ni^II^ and Cu^II^ based complexes fabricated from the sterically crowding cyclo­hexyl-containing Schiff base dyes. Photophysical properties of the reported complexes are also discussed.

## 1. Introduction   

About one and a half centuries ago in his prominent doctoral dissertation, J. D. van der Waals was the first who recognized non-covalent interactions (van der Waals, 1873 ▸). Non-covalent interactions can tentatively be defined as interactions produced during the formation of a molecular cluster upon interaction of atoms or molecules where covalent bonds are neither formed nor broken. Since their first recognition, non-covalent interactions have become greatly important in many areas such as materials, catalysis, synthesis, biomolecules, *etc*. To highlight a pivotal role of this type of interaction it is sufficient to mention that the double-helix structure of DNA is dictated by a bench of non-covalent interactions (Riley & Hobza, 2013 ▸; Watson & Crick, 1953 ▸). Moreover, the importance of non-covalent interactions was further proven by establishing a general/regular series of International Conferences on Non-covalent Interactions (ICNI), with the first one held on 2–6 September 2019 in Lisbon (https://icni2019.eventos.chemistry.pt/). The conference aimed ‘to highlight the role of non-covalent interactions in synthesis, catalysis, crystal engineering, molecular recognition, medicinal chemistry, biology, materials science, electrochemical immobilization, *etc.*, including also theoretical aspects.’

By their physical nature, non-covalent interactions are often classified into main categories, namely dispersion dominated and electrostatic dominated. A third category of non-covalent interactions, where dispersion and electrostatic contributions are comparable, is also often highlighted. Nowadays, non-covalent interactions, depending on the involved atoms or units within molecules, are classified into hydrogen bonding, π⋯π interaction, halogen bonding, chalcogen bonding, tetrel bonding, (an)agostic bonding, cation/anion⋯π interaction and many others (Biedermann & Schneider, 2016 ▸; Hobza & Zahradník, 1988 ▸; Hobza *et al.*, 2006 ▸; Mahadevi & Sastry, 2016 ▸; Müller-Dethlefs & Hobza, 2000 ▸; Řezáč & Hobza, 2016 ▸; Riley & Hobza, 2013 ▸; Riley *et al.*, 2010 ▸). Among the electrostatic and dispersion-dominated non-covalent interactions, the hydrogen bond and the π⋯π interaction, respectively, are the most prominent ones (Biedermann & Schneider, 2016 ▸; Hobza & Zahradník, 1988 ▸; Hobza *et al.*, 2006 ▸; Mahadevi & Sastry, 2016 ▸; Müller-Dethlefs & Hobza, 2000 ▸; Řezáč & Hobza, 2016 ▸; Riley & Hobza, 2013 ▸; Riley *et al.*, 2010 ▸). Notably, non-covalent interactions incorporating aromatic systems are of particular interest owing to their practical applications (Salonen *et al.*, 2011 ▸; Thakuria *et al.*, 2019 ▸; Wheeler, 2013 ▸). The energy of the π⋯π stacking in benzene dimer was calculated to be −2.758 kcal mol^−1^, while the most energetically favourable tilted T-shape interaction gives rise to −2.843 kcal mol^−1^ (Řezáč & Hobza, 2016 ▸). Although the term ‘stacking interaction’ is mainly addressed to aromatic systems, aliphatic systems can also be involved in stacking interactions (Řezáč & Hobza, 2016 ▸). Interestingly, interaction between cyclo­hexane and benzene is more efficient (−3.01 kcal mol^−1^) (Ran & Wong, 2006 ▸) than those in benzene (−2.758 kcal mol^−1^) (Řezáč & Hobza, 2016 ▸) and cyclo­hexane (−2.62 kcal mol^−1^) (Kim *et al.*, 2011 ▸) dimers.

Another peculiar type of non-covalent interaction, namely anagostic interaction (Brookhart *et al.*, 2007 ▸; Sundquist *et al.*, 1990 ▸), is of ever-growing interest owing to its presence in many catalytic processes. This type of interaction is inherent to square-planar *d* ^8^-metal complexes, and sometimes anagostic interactions are speculatively claimed as agostic interactions (Castro *et al.*, 2005 ▸; Thakur & Desiraju, 2006 ▸). However, agostic and anagostic interactions differ significantly from the structural point of view. In particular, the former interactions are characterized by the *M*⋯H distance of ∼1.8–2.2 Å and C—H⋯*M* bond angles of ∼90–140°, while the latter interactions exhibit long *M*⋯H distances of ∼2.3–3.0 Å and C—H⋯*M* bond angles of ∼110–170° (Brookhart *et al.*, 2007 ▸). While agostic bonds are attractive, it is still under debate as to whether anagostic bonds are attractive or repulsive.

Non-covalent interactions were also found to be a powerful tool for crystal engineering of supramolecular structures of coordination compounds (Mahmudov *et al.*, 2017 ▸). Our groups have also extensively been involved in studying non-covalent interactions in the systems of *N*-(thio)­phospho­rylated thio­ureas (Babashkina *et al.*, 2016 ▸, 2011 ▸, 2012 ▸, 2013 ▸; Mitoraj *et al.*, 2018 ▸, 2019*b* ▸,*d* ▸; Safin *et al.*, 2015*a* ▸,*b* ▸, 2014 ▸, 2013*a* ▸,*b* ▸, 2016*a* ▸) and poly *N*-donor compounds (Brunet *et al.*, 2017*a* ▸,*b* ▸; Mahmoudi *et al.*, 2017*a* ▸,*b* ▸,*c* ▸, 2018 ▸; Mitoraj *et al.*, 2019*a* ▸,*c* ▸; Safin *et al.*, 2015*c* ▸, 2017*a* ▸,*b* ▸, 2016*b* ▸), as well as their coordination compounds with metal cations. In particular, we have previously established the crucial influence of non-covalent interactions in crystal engineering of the Ni^II^ complexes with *N*-thio­phospho­rylated thio­ureas *R*NHC(S)NHP(S)(O*i*Pr)_2_ [*R* = (HOCH_2_)(Me)_2_C (Safin *et al.*, 2015*b* ▸), *m*-F_3_CC_6_H_4_ (Mitoraj *et al.*, 2019*b* ▸)]. Notably, we were able to demonstrate for the first time, based on quantum chemical computations, that, depending on the *M*⋯H distance, anagostic interactions can be either repulsive or attractive (Mitoraj *et al.*, 2019*b* ▸). We were also able to demonstrate for the first time, based on quantum chemical computations, that C—H⋯*M* anagostic interactions, despite their long distances (∼3 Å), can be attractive, contrary to the intuitive wisdom (Mitoraj *et al.*, 2019*b* ▸).

**Figure 1:**
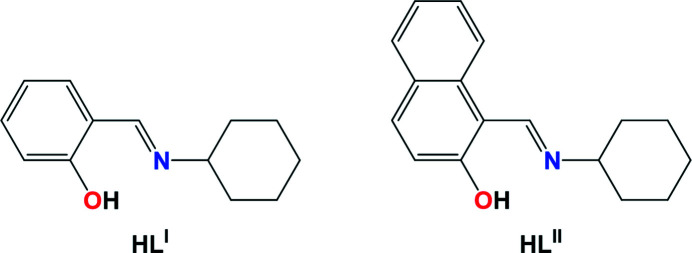
Diagrams of the applied Schiff base dyes.

With all this in mind and in continuation of our investigations in the field of non-covalent interactions, as well as studying their influence on the structure stabilization, we have directed our attention to molecules containing several synthons that can produce non-covalent interactions. Thus, we have addressed Schiff base dyes. The main advantage being the ease of synthesis by condensation of corresponding aldehydes with primary amines under mild conditions. In particular, we have selected bulky *cyclo*­hexyl­amine and salicyaldehyde/2-hy­droxy-1-naphthaldehyde. The resulting Schiff bases *o*-HOC_6_H_4_—CH=N—*cyclo*-C_6_H_11_ (H*L* ^I^) and *o*-HOC_10_H_6_—CH=N—*cyclo*-C_6_H_11_ (H*L* ^II^) (Fig. 1 ▸) were involved in complexation with Ni^II^ and Cu^II^, yielding discrete mononuclear homoleptic complexes [Ni(*L* ^I,II^)_2_] and [Cu(*L* ^I,II^)_2_], respectively. The obtained complexes seem to be excellent platforms to generate a bunch of non-covalent interactions owing to the presence of aromatic benzene rings, aliphatic cyclo­hexane rings and metal-containing chelate rings. Theoretical studies are then applied to shed light on the origin of their photophysical properties. Although the crystal structures of [Ni(*L* ^I^)_2_] (Bhatia *et al.*, 1983 ▸), [Cu(*L* ^I^)_2_] (Jain & Syal, 1988 ▸; Kashyap *et al.*, 1975 ▸; Tamura *et al.*, 1977 ▸) and [Cu(*L* ^II^)_2_] (Fernández-G *et al.*, 1997 ▸) were reported recently, we have decided to redefine the structures with a higher precision as well as identify classic and unintuitive non-covalent interactions responsible for the formation of their supramolecular structures.

## 2. Results and discussion   

A reaction of an equimolar amount of cyclo­hexyl­amine and salicyaldehyde or 2-hy­droxy-1-naphthaldehyde in ethanol under reflux yielded the Schiff bases H*L* ^I,II^ as yellow viscous oil. H*L* ^I,II^ were involved in the reaction with a half molar amount of *M*(CH_3_COO)_2_ (*M* = Ni, Cu) in ethanol. As a result, discrete mononuclear homoleptic complexes [Ni(*L* ^I,II^)_2_] and [Cu(*L* ^I,II^)_2_], respectively, were isolated with high yields.

**Figure 2:**
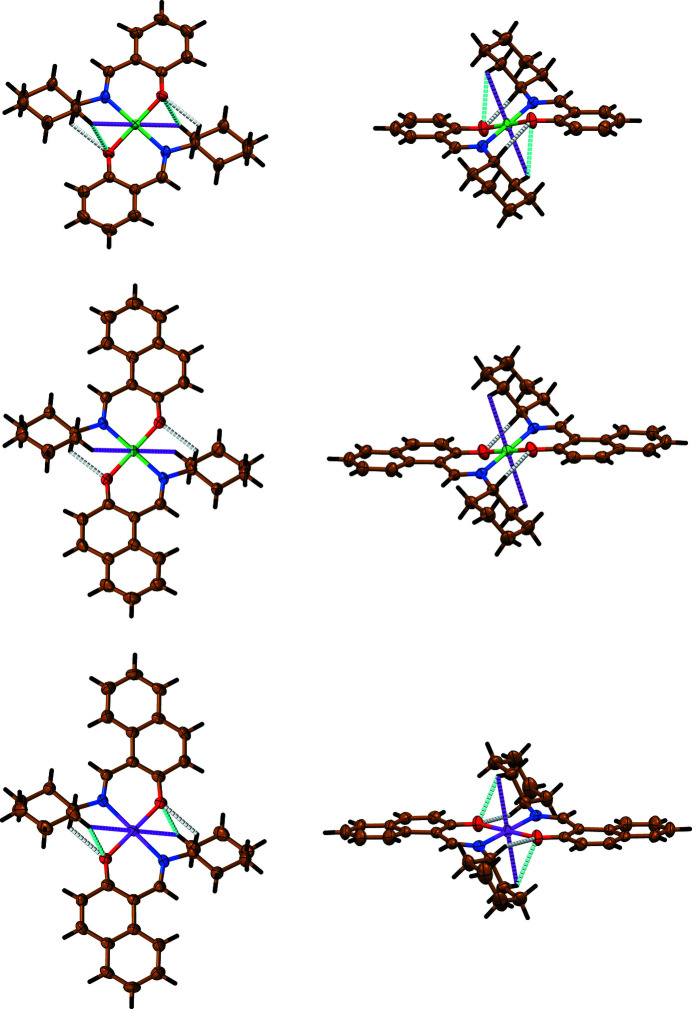
Top and side views of the crystal structures of [Ni(*L* ^I^)_2_] (top row), [Ni(*L* ^II^)_2_] (middle row) and [Cu(*L* ^II^)_2_] (bottom row). Furthermore, 75% atomic displacement ellipsoids are shown for non-hydrogen atoms. Colour code: H = black, C = gold, N = blue, O = red, *M* = green or magenta, an *M*⋯H anagostic bond = magenta dashed line, an O⋯H interaction = grey dashed line and an O⋯H elongated interaction = cyan dashed line . The crystal structure of [Cu(*L* ^I^)_2_] is very similar to that of [Ni(*L* ^I^)_2_].

Complexes [Ni(*L* ^I^)_2_] and [Cu(*L* ^I^)_2_] were found to be isostructural, as shown by the single-crystal X-ray diffraction data (see the *Experimental* Section[Sec sec4]). Their crystal structures were best solved in the triclinic space group *P-*1 (No. 2), while the crystal structures of [Ni(*L* ^II^)_2_] and [Cu(*L* ^II^)_2_] were solved in the monoclinic space group *P*2_1_/*n* with a half of the complex molecule in the asymmetric unit for all the complexes. In complexes, the metal cation is coordinated by two molecules of the deprotonated ligand *L* ^I,II^ via imine nitro­gen atom and phen­oxy oxygen atom affording a tetracoordinate environment with the formation of a perfect square-planar coordination geometry as shown by the τ_4_ descriptor (Fig. 2 ▸, Table 1 ▸) (Yang *et al.*, 2007 ▸). The ligands are linked in a *trans*-configuration with the six-membered chelate rings adopting an envelope conformation in the structures of [Ni(*L* ^I,II^)_2_] and [Cu(*L* ^I^)_2_], while they are much more planar in the structure of [Cu(*L* ^II^)_2_] (Fig. 2 ▸, Table 1 ▸). The cyclo­hexyl fragments are in a typical chair conformation (Fig. 2 ▸). The *M*—N bond lengths are ∼1.9–2.0 Å, while the *M*—O bonds are ∼0.1 Å shorter (Table 1 ▸). The C=N and C—O bonds in the structures of the complexes are very similar and are ∼1.3 Å (Table 1 ▸). Notably, the C=N and C—O bonds are partially double bonds. Both the endo- and exo-cyclic N—*M*—O bond angles are close to 90°, while the N—*M*—N and O—*M*—O angles are 180°. The *M*—N=C and *M*—O—C bond angles are in the range from ∼122 to 131° (Table 1 ▸).

The angles between planes formed by the benzene or naphthyl and cyclo­hexyl rings corresponding to the same ligand in the structures of [Ni(*L* ^I,II^)_2_] and [Cu(*L* ^I^)_2_] are ∼35°, while the same angles in the structure of [Cu(*L* ^II^)_2_] are ∼45°. The same angles between the planes formed by the benzene or naphthalene and chelate rings, and cyclo­hexyl and chelate rings are ∼7–11 and 45°, respectively (Table 1 ▸).

**Figure 3:**
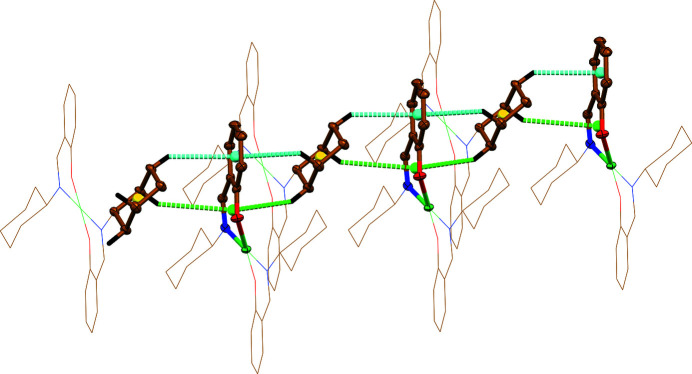
A view on the intermolecular interactions formed by the benzene, cyclo­hexyl and chelate rings in the crystal structures of [Ni(*L* ^I^)_2_] and [Cu(*L* ^I^)_2_] (50% atomic displacement ellipsoids are shown for the non-hydrogen atoms of the interacted fragments). Hydrogen atoms not involved in the interactions are omitted for clarity. Colour code: H = black, C = gold, N = blue, O = red, *M* = green, a C—H⋯π(benzene) interaction = cyan dashed line, a C—H⋯π(chelate) interaction = green dashed line, a centroid of the benzene ring = cyan ball, a centroid of the chelate ring = green ball, a centroid of the cyclo­hexyl ring = yellow ball.

The crystal structures of the complexes are stabilized by a set of intramolecular interactions (Fig. 2 ▸, Table 2 ▸). In particular, the hydrogen atom of the cyclo­hexyl tertiary carbon is involved in the C—H⋯O interaction with the oxygen atom of a second ligand (Fig. 2 ▸, Table 2 ▸). In the structures of [Ni(*L* ^I^)_2_] and [Cu(*L* ^I,II^)_2_] the same oxygen also forms the second C—H⋯O bond with one of the hydrogen atoms from one of the secondary carbons linked to the tertiary carbon (Fig. 2 ▸, Table 2 ▸). However, the latter non-covalent bond is significantly longer than the former one because of the formation of an anagostic bond by the same hydrogen atom (Fig. 2 ▸, Table 2 ▸). The same anagostic bond was found in the structure of [Ni(*L* ^II^)_2_], which formation, together with a coordination geometry of chelate cycles, prevents the formation of the second intramolecular C—H⋯O bond. Notably, all crystal structures are further stabilized by intermolecular non-covalent interactions of the C—H⋯π(benzene/naphthalene) and C—H⋯π(chelate) nature (Fig. 3 ▸, Table 2 ▸).

The bulk samples of all the complexes are free from phase impurities as shown by comparison of the experimental X-ray powder patterns with calculated powder patterns generated from the single-crystal X-ray data (see Fig. S1 in the Supporting information), as well as from the elemental analysis data (see the *Experimental* Section[Sec sec4]).

We further applied a Hirshfeld surface analysis (Spackman & Jayatilaka, 2009 ▸) to study intermolecular interactions in the crystal structures of both complexes. As a result, a set of 2D fingerprint plots (Spackman & McKinnon, 2002 ▸) were generated using *CrystalExplorer 3.1* (Wolff *et al.*, 2012 ▸). In order to estimate the propensity of two chemical species to be in contact, we calculated the enrichment ratios (*E*) (Jelsch *et al.*, 2014 ▸) of the intermolecular contacts.

It was found that the intermolecular H⋯H and H⋯C contacts occupy an overwhelming majority of the molecular surfaces of all the complexes (Table 3 ▸). There is a clear splitting of the H⋯H fingerprint of [Ni(*L* ^I,II^)_2_] and [Cu(*L* ^I^)_2_], which is caused by the shortest contact being between three atoms, rather than being a direct two-atom contact (Figs. S2–S4) (Spackman & McKinnon, 2002 ▸). The H⋯C contacts are shown in the form of ‘wings’ (Figs. S2–S4), with the shortest *d*_e_ + *d*_i_ ≃ 2.7 Å, and are recognized as characteristic of C—H⋯π nature (Spackman & Jayatilaka, 2009 ▸). The structures of [Ni(*L* ^I,II^)_2_] and [Cu(*L* ^I^)_2_] are also characterized by significantly smaller proportions of the H⋯N and H⋯O contacts (Table 3 ▸). Furthermore, the proportions of these contacts are even smaller in the structure of [Cu(*L* ^II^)_2_], while the proportions of the C⋯C, C⋯N, C⋯O and C⋯Cu contacts are quite distinct (Table 3 ▸, Fig. S5). This is explained by the formation of π(chelate)⋯π(naphthalene) intermolecular interactions (Table 2 ▸). Notably, the molecular surface of all the structures is also described by H⋯*M* intermolecular contacts (Table 3 ▸, Figs. S2–S5), which are assigned to the abovementioned intermolecular C—H⋯*M* and C—H⋯π(chelate) interactions (Table 2 ▸). All the H⋯X contacts are favoured in the structures of [Ni(*L* ^I,II^)_2_] and [Cu(*L* ^I^)_2_], since the corresponding enrichment ratios *E*_HX_ are close to or even higher than unity (Table 3 ▸). However, only H⋯H and H⋯C intermolecular contacts are favoured in the structure of [Cu(*L* ^II^)_2_], while remaining contacts are impoverished (Table 3 ▸).

In order to complement the above structural and Hirshfeld surface analyses, and to determine which contacts stabilize/destabilize the obtained crystals, we performed in-depth bonding studies based on the two complementary approaches, namely the charge- and energy-decomposition scheme ETS-NOCV (Mitoraj *et al.*, 2009 ▸) as well as the Interacting Quantum Atoms (IQA) scheme (Blanco *et al.*, 2005 ▸). The former approach is well suited for the description of intermolecular interactions, whereas the latter approach is more convenient for analyses of various intramolecular contacts and is particularly useful since it can determine whether still-controversial long-distance intramolecular C—H⋯*M* contacts could be repulsive (anagostic) or attractive (agostic). We have recently discovered (Mitoraj *et al.*, 2019*b* ▸), contrary to intuition and the recent state of knowledge (Scherer *et al.*, 2015 ▸), that longer C—H⋯Ni distances (∼3 Å) can stabilize the complex structure. However, the shortening of C—H⋯Ni contacts up to ∼2.8 Å, despite amplification of charge delocalizations [Ni(*d*_z__2_) → σ*(C—H)/σ(C—H) → Ni(*d*_z_2)] and London dispersion terms (Lu *et al.*, 2018 ▸), overall might bring the repulsive C—H⋯Ni interactions owing to overwhelming positive (destabilizing) Coulomb constituent (Mitoraj *et al.*, 2019*b* ▸).

**Figure 4:**
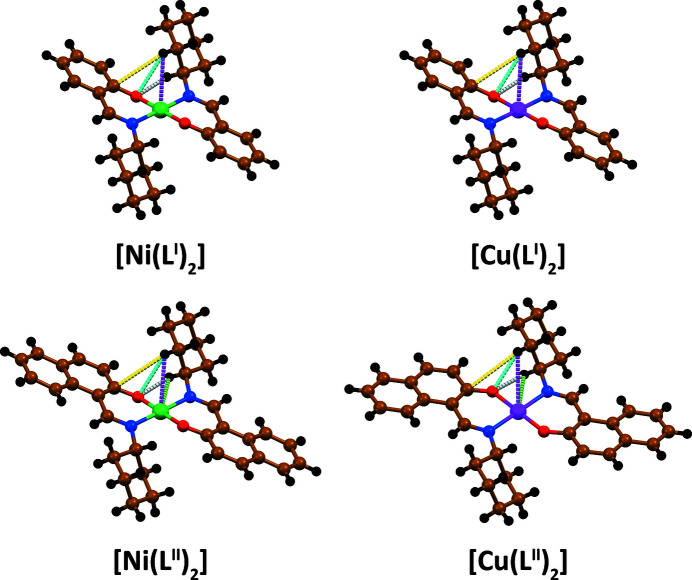
IQA energy decomposition of the selected diatomic interactions obtained at the MP2/6-311 + G(d,p) level of theory for the crystal monomers of [Ni(*L* ^I,II^)_2_] and [Cu(*L* ^I,II^)_2_] (see Table 4 ▸ for details).

The selected IQA/MP2/6-311 + G(d,p) diatomic interaction energies Δ*E*_int_ for the discussed structures are gathered in Fig. 4 ▸ and Table 4 ▸. Notably, despite a long Ni⋯H distance of 2.885 Å in [Ni(*L* ^I^)_2_], a very efficient intramolecular instantaneous stabilization is gained with Δ*E*_int_(Ni⋯H) = −11.36 kcal mol^−1^. It is mainly owing to the attractive Coulomb contribution Δ*E*_Coulomb_ = −10.00 kcal mol^−1^ and slightly stabilizing exchange-correlation term Δ*E*_XC_ = −1.36 kcal mol^−1^ (Fig. 4 ▸, Table 4 ▸). It is important to note that for the Ni^II^ square-planar complex previously studied by us based on *N*-thio­phospho­rylated thio­urea ligands, where exactly the same Ni⋯H distance was noticed (formed by a hydrogen atom of the methyl unit with nickel), the Coulomb term appeared to be positive, which led to the overall repulsive (anagostic) C—H⋯Ni interactions (Mitoraj *et al.*, 2019*b* ▸). This clearly demonstrates different electron-density distribution within the methyl and methyl­ene groups, which in turn is reflected in the opposite values of the Coulomb terms. The origin of such intriguing behaviour will be more carefully studied in the future in order to obtain a more general overview of the nature of long-distance intramolecular C—H⋯*M* interactions.

It was further found that there are two less important stabilizing intramolecular interactions than Ni⋯H: Δ*E*_int_(C⋯H) = −6.99 kcal mol^−1^ and Δ*E*_int_(O⋯H) = −5.89 kcal mol^−1^ (Fig. 4 ▸, Table 4 ▸). The former interaction, belonging to the family of C—H⋯π contacts, is electrostatically dominated with the major attractive Δ*E*_Coulomb_ = −6.41 kcal mol^−1^, whereas, interestingly, in the latter case, the Coulomb term appears to be repulsive and the sole prevailing attractive constituent is the exchange-correlation energy Δ*E*_XC_ = −7.23 kcal mol^−1^ (Fig. 4 ▸, Table 4 ▸). Notably, the second longer O⋯H contact leads to the overall complex destabilization owing to the strongly unfavourable Coulomb contribution, Δ*E*_Coulomb_ = 11.59 kcal mol^−1^, and the weaker exchange-correlation constituent (Fig. 4 ▸, Table 4 ▸). It is a very intriguing physical outcome since C—H⋯O contacts are considered in the literature as rather purely stabilizing interactions (Grabowski, 2011 ▸; Grabowski & Lipkowski, 2011 ▸; Tsuzuki, 2012 ▸). We have shown here that intramolecular C—H⋯O interactions might be both attractive and repulsive depending on distance variation (Fig. 4 ▸, Table 4 ▸). The existence of a stabilizing charge-delocalization channel (XC) for such ultra long distance O⋯H is also an important observation. It has been additionally supported by the ETS-NOCV results where the mentioned intramolecular charge-delocalization channels in addition to C—H⋯H—C (Cukrowski *et al.*, 2016 ▸; Liptrot & Power, 2017 ▸; Mitoraj *et al.*, 2020 ▸; Sagan & Mitoraj, 2019 ▸; Wagner & Schreiner, 2015 ▸) have been discovered (Fig. S6). Recently, the latter has been of particular attention in terms of reconsidering the real nature of steric crowding in bulky species (Cukrowski *et al.*, 2016 ▸; Liptrot & Power, 2017 ▸; Mitoraj *et al.*, 2020 ▸; Sagan & Mitoraj, 2019 ▸; Wagner & Schreiner, 2015 ▸). Notably, substitution of *L* ^I^ by *L* ^II^ leads to a similar picture of the already discussed intramolecular non-covalent interactions (Fig. 4 ▸, Table 4 ▸). Interestingly, in complex [Ni(*L* ^II^)_2_] the second Ni⋯H contact, with quite similar length to the first, was revealed, which, however, destabilizes the overall structure, although quite insignificantly owing to an unfavourable Coulomb term and negligible stemming from the exchange-correlation constituent (Fig. 4 ▸, Table 4 ▸).

As far as the copper-containing complex [Cu(*L* ^I^)_2_] is concerned, quite similar stabilizing intramolecular interactions C⋯H and O⋯H were obtained (Fig. 4 ▸, Table 4 ▸). It is particularly interesting that the Cu⋯H contact is associated with the significant stabilization Δ*E*_int_(Cu⋯H) = −14.16 kcal mol^−1^ despite a very long distance of 3.065 Å (Fig. 4 ▸, Table 4 ▸). Furthermore, the same close contact in [Cu(*L* ^II^)_2_] results in even more efficient preagostic attraction Δ*E*_int_(Cu⋯H) = −14.67 kcal mol^−1^ owing to a shorter distance of 3.015 Å (Fig. 4 ▸, Table 4 ▸). Interestingly, the stabilization in the same complex is further augmented by the second preagostic contact with the corresponding Δ*E*_int_(Cu⋯H) = −8.81 kcal mol^−1^ (Fig. 4 ▸, Table 4 ▸).

**Figure 5:**
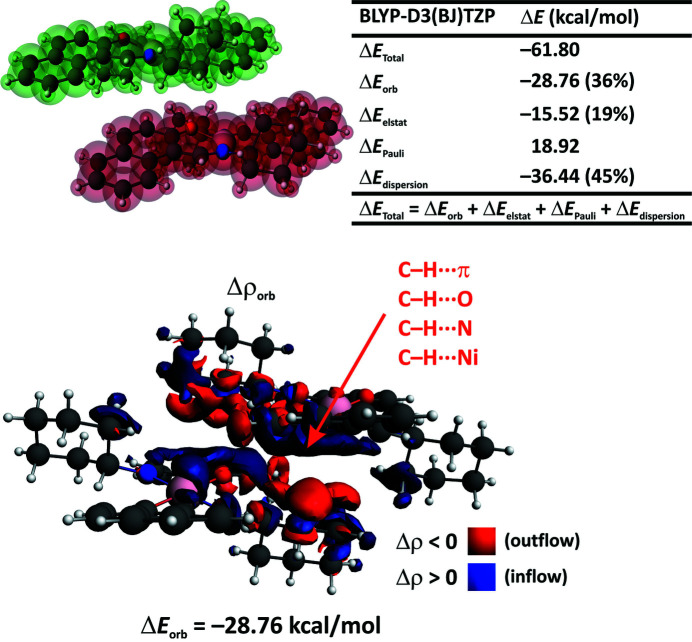
ETS-NOCV/BLYP-D3/TZP energy-decomposition results for the crystal dimer of [Ni(*L* ^II^)_2_]. The considered model and ETS-based results (top), and the overall deformation density Δρ_orb_ with the corresponding Δ*E*_orb_ (bottom).

Finally, we briefly analyzed the intermolecular interactions in the example dimeric model of [Ni(*L* ^II^)_2_] using the ETS-NOCV scheme (Fig. 5 ▸). It was found that the monomers are extremely strongly bonded to each other, with the overall binding energy Δ*E*_total_ = −61.80 kcal mol^−1^ mostly owing to C—H⋯π, C—H⋯O, C—H⋯N and C—H⋯Ni contacts. In line with the literature (Grabowski, 2011 ▸; Grabowski & Lipkowski, 2011 ▸; Tsuzuki, 2012 ▸), the London dispersion constituent is indeed the major contributor with ∼45% of the overall stabilization (Fig. 5 ▸). We have complemented herein that the charge-delocalization contribution Δ*E*_orb_ = −28.76 kcal mol^−1^ is also a crucial cofactor (36% of the overall stabilization) as opposed to the literature claims on insignificance of this constituent (Grabowski, 2011 ▸; Grabowski & Lipkowski, 2011 ▸; Tsuzuki, 2012 ▸). The electrostatic term Δ*E*_elstat_ = −15.52 kcal mol^−1^ appears to be the least important (Fig. 5 ▸). Quite similar sets of intermolecular non-covalent interactions, but significantly weaker, are valid in the counterpart [Ni(*L* ^I^)_2_] (Fig. S7).

**Figure 6:**
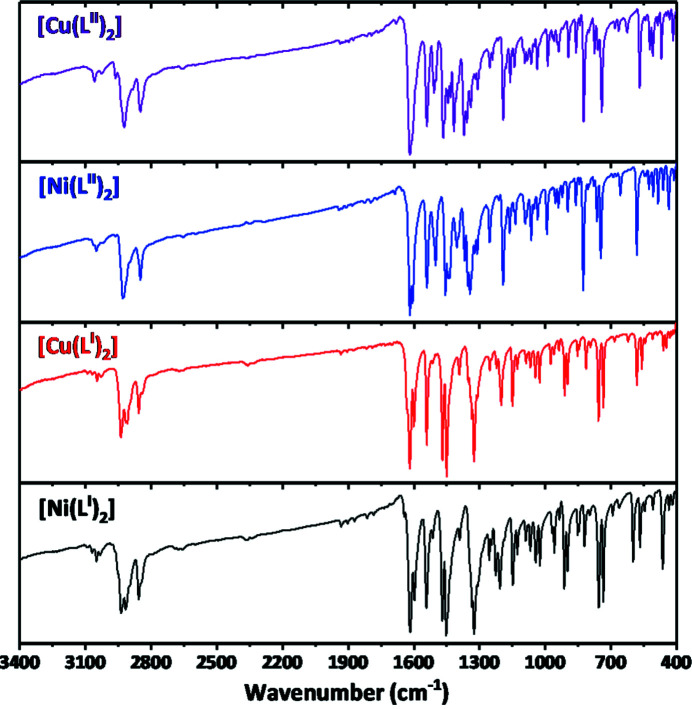
FTIR spectra of [Ni(*L* ^I,II^)_2_] and [Cu(*L* ^I,II^)_2_].

The Fourier transform infrared (FTIR) spectra of the complexes are pairwise very similar and each contain characteristic bands for the C=C and C=N bonds at 1500–1650 cm^−1^ (Fig. 6 ▸). The C—H groups of the cyclo­hexyl fragments are shown as bands at 1325–1340 and 1450 cm^−1^, and a set of bands at 2800–3000 cm^−1^. The aromatic and imine C—H functions are shown as a set of weak bands at 3000–3100 cm^−1^. Notably, the IR spectra of the complexes do not exhibit a characteristic band for the OH group in the range 3200–3400 cm^−1^ (Fig. 6 ▸). This testifies to the deprotonated form of the parent ligands in the structures of the complexes.

**Figure 7:**
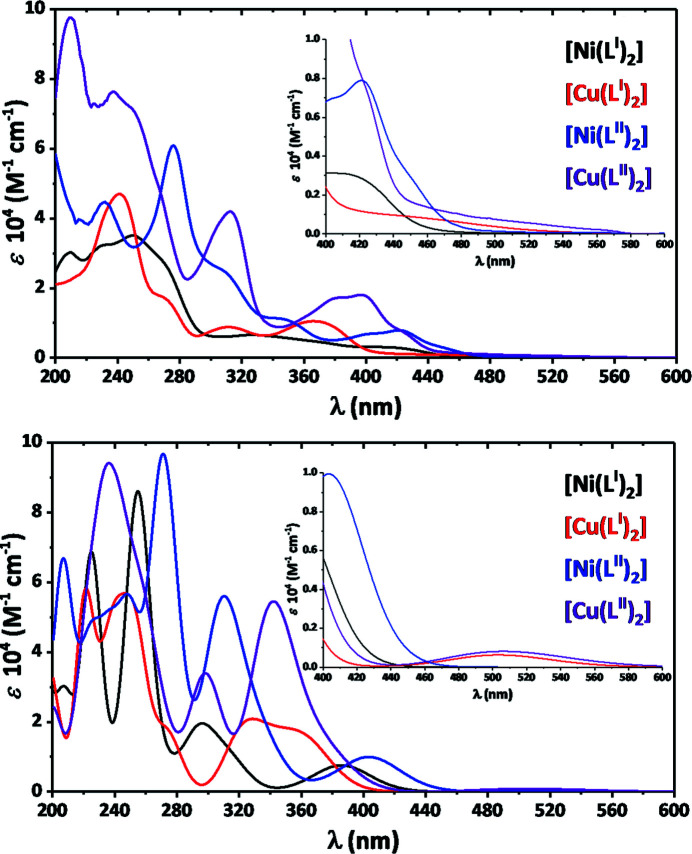
Experimental (top) and simulated (bottom) TDDFT (B3LYP/TZVPP/PCM) UV–Vis absorption spectra of [Ni(*L* ^I,II^)_2_] and [Cu(*L* ^I,II^)_2_] in CH_2_Cl_2_.

Dissolving crystals of [Ni(*L* ^I,II^)_2_] and [Cu(*L* ^I,II^)_2_] in CH_2_Cl_2_ yields yellow and reddish yellow solutions, respectively. In the UV–Vis absorption spectra of the complexes, three regions can be clearly defined. The first region, ranging from 200 to ∼300 nm, contains a set of high intense bands corresponding to intraligand π → π* and *n* → π* transitions (Fig. 7 ▸). The second range at ∼300–440 nm exhibits significantly less intense bands for the metal-to-ligand charge transfer (MLCT) transitions (Fig. 7 ▸). Finally, the weak shoulder in the longer-wavelength region of the spectra is caused by ligand field (*d*–*d*) transitions (Fig. 7 ▸).

**Figure 8:**
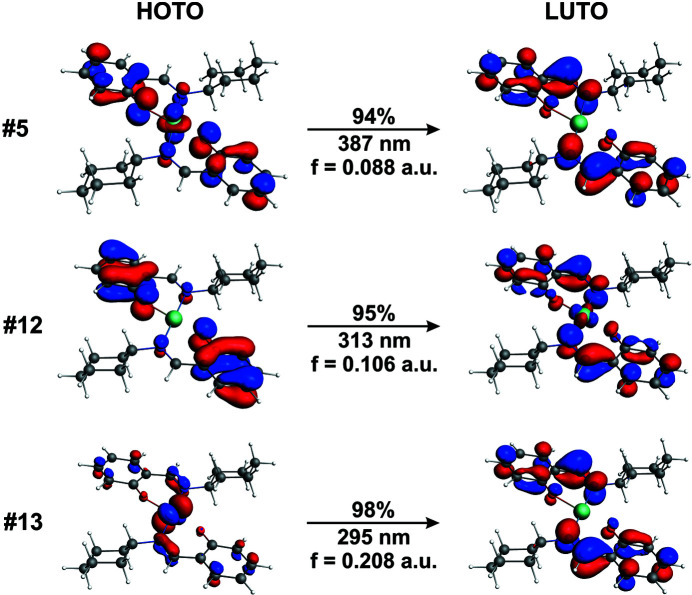
Isosurfaces (±0.04 a.u.) of dominant NTO (natural transition orbital) pairs for the selected excited states of [Ni(*L* ^I^)_2_] along with the percentage weights of hole–particle, corresponding S_0_ → S_1_ transition wavelengths and oscillator strengths (*f*). HOTO = highest occupied transition orbital, LUTO = lowest occupied transition orbital.

In order to shed light on the electronic transitions, we reoptimized all four complexes followed by modelling of the absorption spectra with the TDDFT/B3LYP/TZVPP/PCM(CH_2_Cl_2_) calculations. Since the qualitative picture of the electronic transitions is similar for all the complexes, we briefly discuss the data for [Ni(*L* ^I^)_2_]. All the complexes remain a square-planar geometry in CH_2_Cl_2_ and, in line with the experimental data, the analogous three absorption regions were obtained for all species (Fig. 7 ▸). The absorption bands at 300–400 nm are indeed predominantly characterized as MLCT, *d*_*xz*_(*M*) → π*, as indicated by the dominant transition #13 with the oscillator strength *f* = 0.208 a.u. (Fig. 8 ▸). However, the latter two less intense transitions, #12 (*f* = 0.106 a.u.) and #5 (*f* = 0.088 a.u.), are additionally described by both the ligand-to-ligand and ligand-to-metal charge transfers (Fig. 8 ▸).

**Figure 9:**
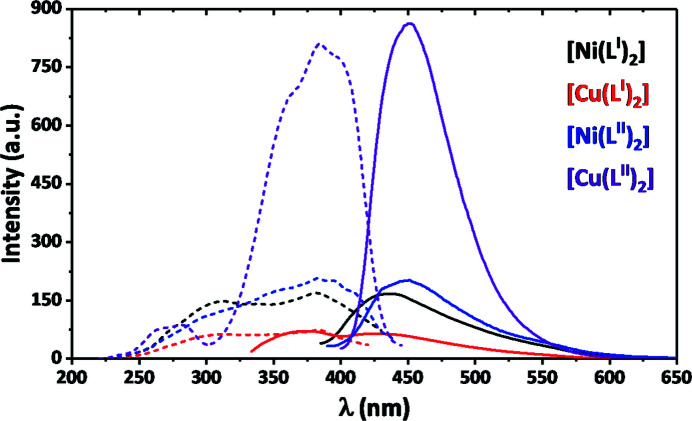
Emission {straight line; λ_exc_ = 380 nm for [Ni(*L* ^I,II^)_2_] and [Cu(*L* ^II^)_2_], and 310 nm for [Cu(*L* ^I^)_2_]} and excitation {dashed line; λ_em_ = 435 nm for [Ni(*L* ^I^)_2_] and [Cu(*L* ^I^)_2_], and 450 nm for [Ni(*L* ^II^)_2_] and [Cu(*L* ^II^)_2_]} spectra for the reported complexes in CH_2_Cl_2_.

Importantly, it was found that all the complexes are emissive in CH_2_Cl_2_; however, complex [Cu(*L* ^II^)_2_] is remarkably more emissive (Fig. 9 ▸). The emission spectra of [Ni(*L* ^I,II^)_2_] and [Cu(*L* ^II^)_2_] exhibit a broad intense band centred at ∼435–450 nm, while the spectrum of [Cu(*L* ^I^)_2_] exhibits a broad band with two maxima at ∼375 and 430 nm (Fig. 9 ▸). Assignment of these bands was made based on the excitation spectra (Fig. 9 ▸). As evident from comparison of the excitation and UV–Vis spectra of the complexes, the emission bands arise from the MLCT emission.

## 3. Conclusions   

In summary, we studied structural and photophysical properties of the Ni^II^ and Cu^II^ discrete mononuclear homoleptic complexes [Ni(*L* ^I,II^)_2_] and [Cu(*L* ^I,II^)_2_], fabricated from the Schiff base dyes *o*-HOC_6_H_4_—CH=N—*cyclo*-C_6_H_11_ (H*L* ^I^) and *o*-HOC_10_H_6_—CH=N—*cyclo*-C_6_H_11_ (H*L* ^II^), respectively, each containing a bulky aliphatic fragment, namely cyclo­hexyl.

Single-crystal X-ray diffraction revealed that all the structures exhibit a *trans*-square-planar geometry. Remarkably, the six-membered metallocycles adopt a clearly defined envelope conformation in [Ni(*L* ^I,II^)_2_] and [Cu(*L* ^I^)_2_], while they are much more planar in the structure of [Cu(*L* ^II^)_2_]. This was found to be clearly associated with the formation of different intra- and inter-molecular contacts, which were deeply characterized by the charge- and energy-decomposition scheme ETS-NOCV as well as the IQA approach. In particular, London dispersion dominated intramolecular C—H⋯O, C—H⋯N and C—H⋯H—C interactions were identified and, predominantly, the attractive, mostly Coulomb driven, C—H⋯Ni/Cu preagostic (not repulsive anagostic) bonds were discovered despite their long distances (∼2.8–3.1 Å). Interestingly, despite the long distances, non-negligible charge-delocalization constituent was discovered. Notably, all the crystal structures are further stabilized by very efficient (the interaction energy is >60 kcal mol^−1^) intermolecular C—H⋯π(benzene) and C—H⋯π(chelate) interactions, which are responsible for their high stability as seen from the thermogravimetric (TG) analyses. Although they contain the prevailing dispersion constituent, the charge-delocalization contribution is only slightly less important followed by the Coulomb term. Our results, clearly showing that the bulky cyclo­hexyl groups are the sources of London dispersion stabilization, are in line with the recent discoveries outlining the true character of steric effects in small and sizable species (Cukrowski *et al.*, 2016 ▸; Liptrot & Power, 2017 ▸; Mitoraj *et al.*, 2019*d* ▸, 2020 ▸; Sagan & Mitoraj, 2019 ▸; Wagner & Schreiner, 2015 ▸). Furthermore, we have determined that intramolecular C—H⋯O interactions can be both attractive and repulsive depending on the distance.

Finally, dissolving crystals of the complexes in CH_2_Cl_2_ yielded yellow and reddish yellow solutions for the Ni^II^ and Cu^II^ derivatives, respectively. The UV–Vis absorption spectra exhibit three clearly defined regions, corresponding to intraligand π → π* and *n* → π* transitions, MLCT transitions and ligand field (*d*–*d*) transitions, as indicated by the time-dependent density functional theory (TDDFT) computations. Importantly, all the complexes were found to be planar and photoluminescent in CH_2_Cl_2_, with [Cu(*L* ^II^)_2_] exhibiting the most pronounced emission, mostly owing to MLCT transitions.

## 4. Experimental   

### 4.1. Materials   

All reagents and solvents were commercially available and used without further purification.

### 4.2. Physical measurements   

Nuclear magnetic resonance (NMR) spectra in CDCl_3_ were obtained on a Bruker AVANCE II 400 MHz spectrometer at 25°C. Chemical shifts are reported with reference to SiMe_4_. Infrared spectra (KBr) were recorded with a FT-IR FSM 1201 spectrometer in the range 400–3400 cm^–1^. UV–Vis and fluorescent spectra from the freshly prepared solutions (5 × 10^−5^ *M*) in freshly distilled CH_2_Cl_2_ were recorded on an Agilent 8453 instrument and a RF-5301 spectro­fluoro­photometer. TG analyses were performed by a NETZSCH STA 449 F5 Jupiter instrument in a dynamic air or argon atmosphere (100 ml min^−1^) from laboratory temperature to 1000°C with a 10°C min^−1^ heating rate. Microanalyses were performed using a ElementarVario EL III analyzer.

### 4.3. Synthesis of H*L* ^I,II^   

A solution of an equimolar amount of salicyl­aldehyde or 2-hy­droxy-1-naphthaldehyde (10 mmol; 1.221 and 1.722 g, respectively) and cyclo­hexyl­amine (10 mmol, 0.992 g) in ethanol (50 ml) was stirred for 1 h under reflux. For a solution of H*L* ^I^, the solvent and non-reacted starting materials were removed *in vacuo*. The resulting yellow viscous oil was analyzed and used as is. The resulting solution of H*L* ^II^ was allowed to cool to room temperature to give crystals, which were filtered off.

(*a*) H*L* ^I^. Yield = 1.809 g (89%). ^1^H NMR: δ = 1.28–1.75 (*m*, 6H, CH_2_, C_6_H_11_), 1.80–1.95 (*m*, 4H, CH_2_, C_6_H_11_), 3.25–3.35 (*m*, 1H, CH, C_6_H_11_), 6.84 (*t*, ^3^ *J*_H,H_ = 7.4 Hz, 1H, 5-H, C_6_H_4_), 6.96 (*d*, ^3^ *J*_H,H_ = 8.2 Hz, 1H, 3-H, C_6_H_4_), 7.32 (*d*, ^3^ *J*_H,H_ = 7.4 Hz, 1H, 6-H, C_6_H_4_), 7.38 (*d*, ^3^ *J*_H,H_ = 8.3 Hz, 1H, 4-H, C_6_H_4_), 8.37 (*s*, 1H, imine) and 13.30 (br. *s*, 1H, OH). Analysis calculated for C_13_H_17_NO (203.29): C = 76.81, H = 8.43 and N = 6.89%; found: C = 76.68, H = 8.37 and N = 6.94%.

(*b*) H*L* ^II^. Yield = 2128 g (84%). ^1^H NMR: δ = 1.30–1.76 (*m*, 6H, CH_2_, C_6_H_11_), 1.86–1.97 (*m*, 2H, CH_2_, C_6_H_11_), 2.03–2.12 (*m*, 2H, CH_2_, C_6_H_11_), 3.44–3.58 (*m*, 1H, CH, C_6_H_11_), 6.94 (*d*, ^3^ *J*_H,H_ = 9.8 Hz, 1H, C_10_H_6_), 7.26 (*t*, ^3^ *J*_H,H_ = 7.3 Hz, 1H, C_10_H_6_), 7.46 (*t*, ^3^ *J*_H,H_ = 7.3 Hz, 1H, C_10_H_6_), 7.64 (*d*, ^3^ *J*_H,H_ = 7.8 Hz, 1H, C_10_H_6_), 7.71 (*d*, ^3^ *J*_H,H_ = 9.8 Hz, 1H, C_10_H_6_), 7.87 (*d*, ^3^ *J*_H,H_ = 7.8 Hz, 1H, C_10_H_6_), 8.77 [*d*, ^3^ *J*_H,H_ = 5.9 Hz, 1H, (naphthalene)CHN], 14.58 (br. *s*, 1H, NH). Analysis calculated for C_17_H_19_NO (253.35): C = 80.60, H = 7.56 and N = 5.53%; found: C = 80.48, H = 7.62 and N = 5.48%.

### 4.4. Synthesis of [Ni(*L* ^I,II^)_2_] and [Cu(*L* ^I,II^)_2_]   

To a solution of H*L* ^I,II^ (2 mmol; 0.407 and 0.507 g, respectively) in ethanol (10 ml) was added a solution of Ni(CH_3_COO)_2_4H_2_O (0.249 g, 1 mmol) or Cu(CH_3_COO)_2_ (0.182 g, 1 mmol) in a mixture of water (1 ml) and ethanol (50 ml). The mixture was stirred at room temperature for 1 h. The resulting precipitate was filtered off, washed with ethanol (3 × 50 ml) and dried *in vacuo*. Then the product was dissolved in CH_2_Cl_2_. X-ray suitable crystals were formed during the next few days upon slow evaporation of the solvent.

(i) [Ni(*L* ^I^)_2_]. Light brown needle-like crystals. Yield = 0.389 g (84%). Analysis calculated for C_26_H_32_N_2_NiO_2_ (463.25): C = 67.41, H = 6.96 and N = 6.05%; found: C = 67.52, H = 7.05 and N = 5.97%.

(ii) [Cu(*L* ^I^)_2_]. Dark red block-like crystals. Yield = 0.360 g (77%). Analysis calculated for C_26_H_32_CuN_2_O_2_ (468.10): C = 66.71, H = 6.89 and N = 5.98%; found: C = 66.62, H = 6.79 and N = 5.91%.

(iii) [Ni(*L* ^II^)_2_]. Green needle-like crystals. Yield = 0.439 g (78%). Analysis calculated for C_34_H_36_N_2_NiO_2_ (563.37): C = 72.49, H = 6.44 and N = 4.97%; found: C = 72.61, H = 6.49 and N = 5.02%.

(iv) [Cu(*L* ^II^)_2_]. Greenish yellow needle-like crystals. Yield = 0.472 g (83%). Analysis calculated for C_34_H_36_CuN_2_O_2_ (568.22): C = 71.87, H = 6.39 and N = 4.93%; found: C = 71.98, H = 6.34 and N = 4.88%.

### 4.5. X-ray powder diffraction of [Ni(*L* ^I,II^)_2_] and [Cu(*L* ^I,II^)_2_]   

X-ray powder diffraction for a bulk sample was carried out using a Rigaku Miniflex X-ray powder diffractometer (λ = 1.54059 Å).

### 4.6. Single-crystal X-ray diffraction of [Ni(*L* ^I,II^)_2_] and [Cu(*L* ^I,II^)_2_]   

Data for all the structures were collected on a Stoe IPDS II two-circle diffractometer with a Genix Microfocus tube with mirror optics using Mo *K*α radiation (λ = 0.71073 Å). The data were scaled using the frame-scaling procedure in the *X-AREA* program system (Stoe & Cie, 2002 ▸). The structures were solved by direct methods using the program *SHELXS* (Sheldrick, 2008 ▸, 2015 ▸) and refined against *F* ^2^ with full-matrix least-squares techniques using the program *SHELXL* (Sheldrick, 2008 ▸, 2015 ▸). Hydrogen atoms were geometrically positioned and refined using a riding model.

(1) Crystal data for [Ni(*L* ^I^)_2_]. C_26_H_32_N_2_NiO_2_, *M*_r_ = 463.24 g mol^−1^, *T* = 173 (2) K, triclinic, space group *P-*1 (No. 2), *a* = 6.4256 (6), *b* = 7.7129 (8), *c* = 11.9856 (11) Å, α = 98.709 (8), β = 101.800 (8), γ = 104.300 (8)°, *V* = 550.52 (10) Å^3^, *Z* = 1, ρ = 1.397 g cm^−3^, μ(Mo *K*α) = 0.907 mm^−1^, reflections = 6716 collected and 2530 unique, *R*_int_ = 0.0361, *R*_1_(all) = 0.0592, *wR*_2_(all) = 0.1094 and *S* = 1.151.

(2) Crystal data for [Cu(*L* ^I^)_2_]. C_26_H_32_CuN_2_O_2_, *M*_r_ = 468.07 g mol^−1^, *T* = 173 (2) K, triclinic, space group *P-*1 (No. 2), *a* = 6.4641 (4), *b* = 7.7224 (5), *c* = 11.9925 (7) Å, α = 97.647 (5), β = 101.861 (5), γ = 105.261 (5)°, *V* = 553.99 (6) Å^3^, *Z* = 1, ρ = 1.403 g cm^−3^, μ(Mo *K*α) = 1.011 mm^−1^, reflections = 12 420 collected and 3073 unique, *R*_int_ = 0.0206, *R*_1_(all) = 0.0252, *wR*_2_(all) = 0.0693 and *S* = 1.103.

(3) Crystal data for [Ni(*L* ^II^)_2_]. C_34_H_36_N_2_NiO_2_, *M*_r_ = 563.36 g mol^−1^, *T* = 173 (2) K, monoclinic, space group *P*2_1_/*n*, *a* = 6.0847 (3), *b* = 10.5704 (7), *c* = 20.8597 (11) Å, β = 97.882 (4)°, *V* = 1328.97 (13) Å^3^, *Z* = 2, ρ = 1.408 g cm^−3^, μ(Mo *K*α) = 0.766 mm^−1^, reflections = 15 012 collected and 2930 unique, *R*_int_ = 0.043, *R*_1_(all) = 0.0527, *wR*_2_(all) = 0.0879 and *S* = 1.106.

(4) Crystal data for [Cu(*L* ^II^)_2_]. C_34_H_36_CuN_2_O_2_, *M*_r_ = 568.19 g mol^−1^, *T* = 173 (2) K, monoclinic, space group *P*2_1_/*n*, *a* = 11.0325 (10), *b* = 5.6889 (3), *c* = 21.554 (2) Å, β = 99.410 (7)°, *V* = 1334.59 (19) Å^3^, *Z* = 2, ρ = 1.414 g cm^−3^, μ(Mo *K*α) = 0.854 mm^−1^, reflections = 10 452 collected, 2485 unique, *R*_int_ = 0.032, *R*_1_(all) = 0.0455, *wR*_2_(all) = 0.0842 and *S* = 1.137.

## Supplementary Material

Crystal structure: contains datablock(s) ba37, ba36, ba43, ba41. DOI: 10.1107/S2052252521000610/ed5022sup1.cif

Supporting information. DOI: 10.1107/S2052252521000610/ed5022sup2.pdf

CCDC references: 1959601, 1972277, 1972278, 2056955

## Figures and Tables

**Table 1:** Selected bond lengths (Å) and angles (°) for [Ni(*L* ^I,II^)_2_] and [Cu(*L* ^I,II^)_2_]

	[Ni(*L* ^I^)_2_]	[Cu(*L* ^I^)_2_]	[Ni(*L* ^II^)_2_]	[Cu(*L* ^II^)_2_]
Bond lengths (Å)				
*M*—N	1.943 (2)	2.0184 (9)	1.924 (2)	2.0236 (18)
*M*—O	1.845 (2)	1.8969 (9)	1.830 (1)	1.894 (2)
C=N	1.288 (3)	1.2902 (14)	1.297 (2)	1.289 (3)
C—O	1.313 (3)	1.3089 (14)	1.306 (2)	1.297 (3)
Bond angles (°)				
N—*M*—O_endocyclic_	91.30 (9)	90.58 (4)	90.73 (7)	90.06 (7)
N—*M*—O_exocyclic_	88.71 (9)	89.42 (4)	89.27 (7)	89.94 (7)
N—*M*—N	180.00	180.00	180.00	180.00
O—*M*—O	180.00	180.00	180.00	180.00
*M*—N=C	122.77 (17)	121.86 (8)	123.59 (14)	123.86 (15)
*M*—O—C	125.28 (16)	125.74 (8)	127.04 (12)	131.43 (15)
Torsion angles (°)†				
*M*—N=C—C_chelate_	8.2 (4)	8.86 (16)	−5.2 (3)	−7.1 (3)
*M*—O—C—C_chelate_	−26.8 (3)	−25.23 (17)	22.5 (3)	2.2 (3)
N—*M*—O—C_chelate_	36.5 (2)	34.25 (10)	−33.05 (16)	−9.6 (2)
O—*M*—N=C_chelate_	−26.9 (2)	−25.60 (10)	23.97 (17)	11.37 (18)
N=C—C—C_chelate_	12.8 (4)	11.39 (19)	−15.7 (3)	−4.0 (4)
O—C—C—C_chelate_	−3.8 (4)	−3.64 (18)	7.5 (3)	7.1 (3)
Angles between planes				
aryl⋯*cyclo*-C_6_H_11_	34.41 (13)	33.96 (6)	35.37 (8)	44.76 (10)
aryl⋯*M*NCCCO	10.06 (12)	10.21 (5)	10.94 (6)	7.19 (7)
*cyclo*-C_6_H_11_⋯*M*NCCCO	43.64 (12)	43.49 (5)	44.51 (9)	45.11 (11)

† Torsion angles must be compared by their magnitudes.

**Table 2:** Selected non-covalent bond lengths (Å) and angles (°) for [Ni(*L* ^I,II^)_2_] and [Cu(*L* ^I,II^)_2_]

	D—H⋯A (Å)	*d*(D—H) (Å)		*d*(H⋯A) (Å)		*d*(D⋯A) (Å)		∠(DHA) (°)
[Ni(*L* ^I^)_2_]	C—H⋯O	1.00		2.25		2.770 (3)		111
	C—H⋯O	0.99		2.66		3.165 (4)		112
	C—H⋯Ni	0.99		2.88		3.385 (3)		112
	C—H⋯π(benzene)	0.99		2.92		3.740 (3)		140
	C—H⋯π(benzene)	0.99		2.91		3.753 (3)		144
	C—H⋯π(chelate)	0.99		2.74		3.64		151
	C—H⋯π(chelate)	0.99		3.29		4.12		143
[Cu(*L* ^I^)_2_]	C—H⋯O	1.00		2.31		2.845 (2)		112
	C—H⋯O	0.99		2.67		3.195 (2)		113
	C—H⋯Cu	0.99		2.91		3.432 (1)		114
	C—H⋯π(benzene)	0.99		2.89		3.711 (2)		141
	C—H⋯π(benzene)	0.99		2.86		3.722 (2)		146
	C—H⋯π(chelate)	0.99		2.71		3.60		150
	C—H⋯π(chelate)	0.99		3.23		4.08		145
[Ni(*L* ^II^)_2_]	C—H⋯O	1.00		2.19		2.733 (2)		113
	C—H⋯Ni	0.99		2.90		3.395 (2)		112
	C—H⋯π(C_6_H_2_)	0.99		2.81		3.741 (2)		157
	C—H⋯π(C_6_H_4_)	0.99		2.73		3.645 (2)		154
	C—H⋯π(chelate)	0.99		2.91		3.68		135
[Cu(*L* ^II^)_2_]	C—H⋯O	1.00		2.30		2.790 (3)		109
	C—H⋯O	0.99		2.45		3.012 (3)		115
	C—H⋯Cu	0.99		3.02		3.512 (2)		112
	C—H⋯π(C_6_H_4_)	0.95		2.69		3.466 (2)		140
	C—H⋯π(C_6_H_4_)	0.99		2.74		3.674 (2)		157
			*d*(Cg–Cg) (Å)		∠(Cg–Cg) (°)		Slippage (Å)	
	π(chelate)⋯π(C_6_H_2_)		3.9578 (13)		5.80 (9)		2.163	
	π(chelate)⋯π(C_10_H_6_)		4.0692 (11)		7.19 (7)		2.229	

**Table 3:** Hirshfeld contact surfaces, derived ‘random contacts’ and ‘enrichment ratios’ for [Ni(*L* ^I,II^)_2_] and [Cu(*L* ^I,II^)_2_] Fingerprint plots of the observed contacts are available in the Supporting information.

	[Ni(*L* ^I^)_2_]					[Cu(*L* ^I^)_2_]					[Ni(*L* ^II^)_2_]					[Cu(*L* ^II^)_2_]				
	H	C	N	O	Ni	H	C	N	O	Cu	H	C	N	O	Ni	H	C	N	O	Cu
Contacts (*C*, %)†																				
H	67.2	–	–	–	–	66.3	–	–	–	–	58.8	–	–	–	–	62.2	–	–	–	–
C	23.9	0.0	–	–	–	24.3	0.0	–	–	–	33.5	0.1	–	–	–	28.9	1.1	–	–	–
N	2.0	0.2	0.0	–	–	2.1	0.2	0.0	–	–	2.3	0.0	0.0	–	–	1.1	1.5	0.0	–	–
O	5.6	0.0	0.0	0.0	–	5.5	0.0	0.0	0.0	–	4.8	0.0	0.0	0.0	–	0.9	1.6	0.0	0.4	–
*M*	1.2	0.0	0.0	0.0	0.0	1.6	0.0	0.0	0.0	0.0	0.5	0.0	0.0	0.0	0.0	0.8	1.5	0.0	0.0	0.0
Surface (*S*, %)																				
	83.6	12.1	1.1	2.8	0.6	83.1	12.3	1.2	2.8	0.8	79.4	16.9	1.2	2.4	0.3	78.1	17.9	1.3	1.7	1.2
Random contacts (*R*, %)																				
H	69.9	–	–	–	–	69.1	–	–	–	–	63.0	–	–	–	–	61.0	–	–	–	–
C	20.2	1.5	–	–	–	20.4	1.5	–	–	–	28.8	2.9	–	–	–	28.0	3.2	–	–	–
N	1.8	0.3	0.0	–	–	2.0	0.3	0.0	–	–	1.9	0.4	0.0	–	–	2.0	0.5	0.0	–	–
O	4.7	0.7	0.1	0.1	–	4.7	0.7	0.1	0.1	–	3.8	0.8	0.1	0.1	–	2.7	0.6	0.0	0.0	–
*M*	1.0	0.1	0.0	0.0	0.0	1.3	0.2	0.0	0.0	0.0	0.5	0.1	0.0	0.0	0.0	1.9	0.4	0.0	0.0	0.0
Enrichment (*E*)‡																				
H	0.96	–	–	–	–	0.96	–	–	–	–	0.93	–	–	–	–	1.02	–	–	–	–
C	1.18	0.0	–	–	–	1.19	0.0	–	–	–	1.16	0.03	–	–	–	1.03	0.34	–	–	–
N	1.11	–	–	–	–	1.05	–	–	–	–	1.21	–	–	–	–	0.55	–	–	–	–
O	1.19	–	–	–	–	1.17	–	–	–	–	1.26	–	–	–	–	0.33	–	–	–	–
*M*	1.20	–	–	–	–	1.23	–	–	–	–	–	–	–	–	–	0.42	–	–	–	–

† Values were obtained from *CrystalExplorer 3.1* (Wolff *et al.*, 2012 ▸).

‡ The enrichment ratios were not computed when the random contacts were lower than 0.9%, as they are not meaningful (Jelsch *et al.*, 2014 ▸).

**Table 4:** IQA energy decomposition of the selected diatomic interactions obtained at the MP2/6-311 + G(d,p) level of theory for the crystal monomers of [Ni(*L* ^I,II^)_2_] and [Cu(*L* ^I,II^)_2_] Δ*E*_int_ = Δ*E*_Coulomb_ + Δ*E*_XC_, where Δ*E*_int_ is the overall diatomic interaction energy, Δ*E*_Coulomb_ is the Coulomb constituent and Δ*E*_XC_ is the exchange-correlation contribution (Blanco *et al.*, 2005 ▸).

	*d*(A⋯B) (Å)	Δ*E*_int_^AB^ (kcal mol^−1^)	Δ*E*_Coulomb_ (kcal mol^−1^)	Δ*E*_XC_ (kcal mol^−1^)
[Ni(*L* ^I^)_2_]				
Ni⋯H (magenta dashed line)	2.885	−11.36	−10.00	−1.36
O⋯H (grey dashed line)	2.247	−5.89	1.34	−7.23
O⋯H (cyan dashed line)	2.660	8.90	11.59	−2.70
C⋯H (yellow dashed line)	3.033	−6.99	−6.41	−0.58
[Cu(*L* ^I^)_2_]				
Cu⋯H (magenta dashed line)	3.065	−14.16	−13.30	−0.86
O⋯H (grey dashed line)	2.309	−4.24	2.15	−6.39
O⋯H (cyan dashed line)	2.671	10.60	13.46	−2.86
C⋯H (yellow dashed line)	3.118	−7.61	−7.21	−0.40
[Ni(*L* ^II^)_2_]				
Ni⋯H (magenta dashed line)	2.901	−11.77	−10.44	−1.33
Ni⋯H (green dashed line)	2.975	0.52	0.65	−0.13
O⋯H (grey dashed line)	2.186	−6.84	1.34	−8.17
O⋯H (cyan dashed line)	2.696	9.53	11.93	−2.40
C⋯H (yellow dashed line)	3.138	−7.10	−6.71	−0.40
[Cu(*L* ^II^)_2_]				
Cu⋯H (magenta dashed line)	3.015	−14.67	−14.22	−0.44
Cu⋯H (green dashed line)	3.017	−8.81	−8.69	−0.13
O⋯H (grey dashed line)	2.295	5.01	11.06	−6.05
O⋯H (cyan dashed line)	2.454	10.52	14.85	−4.63
C⋯H (yellow dashed line)	3.033	−9.68	−9.55	−0.13
