# Supplementary material for: Supramolecular structures of NiII and CuII with the sterically demanding Schiff base dyes driven by cooperative action of preagostic and other non-covalent interactions
Source: IUCrJ. 2021 May 1;8(Pt 3):351–61. doi: 10.1107/S2052252521000610 (PMC8086159; doi:10.1107/S2052252521000610)
Supplement: Supplementary file 2 [file m-08-00351-sup2.pdf]

IUCrJ

Volume 8 (2021)

Supporting information for article:

Supramolecular structures of Ni^{II} and Cu^{II} with the sterically demanding Schiff base dyes driven by cooperative action of preagostic and other non-covalent interactions

Alexey A. Shiryaev, Tatyana M. Burkhanova, Mariusz P. Mitoraj, Mercedes Kukulka, Filip Sagan, Ghodrat Mahmoudi, Maria G. Babashkina, Michael Bolte and Damir A. Safin

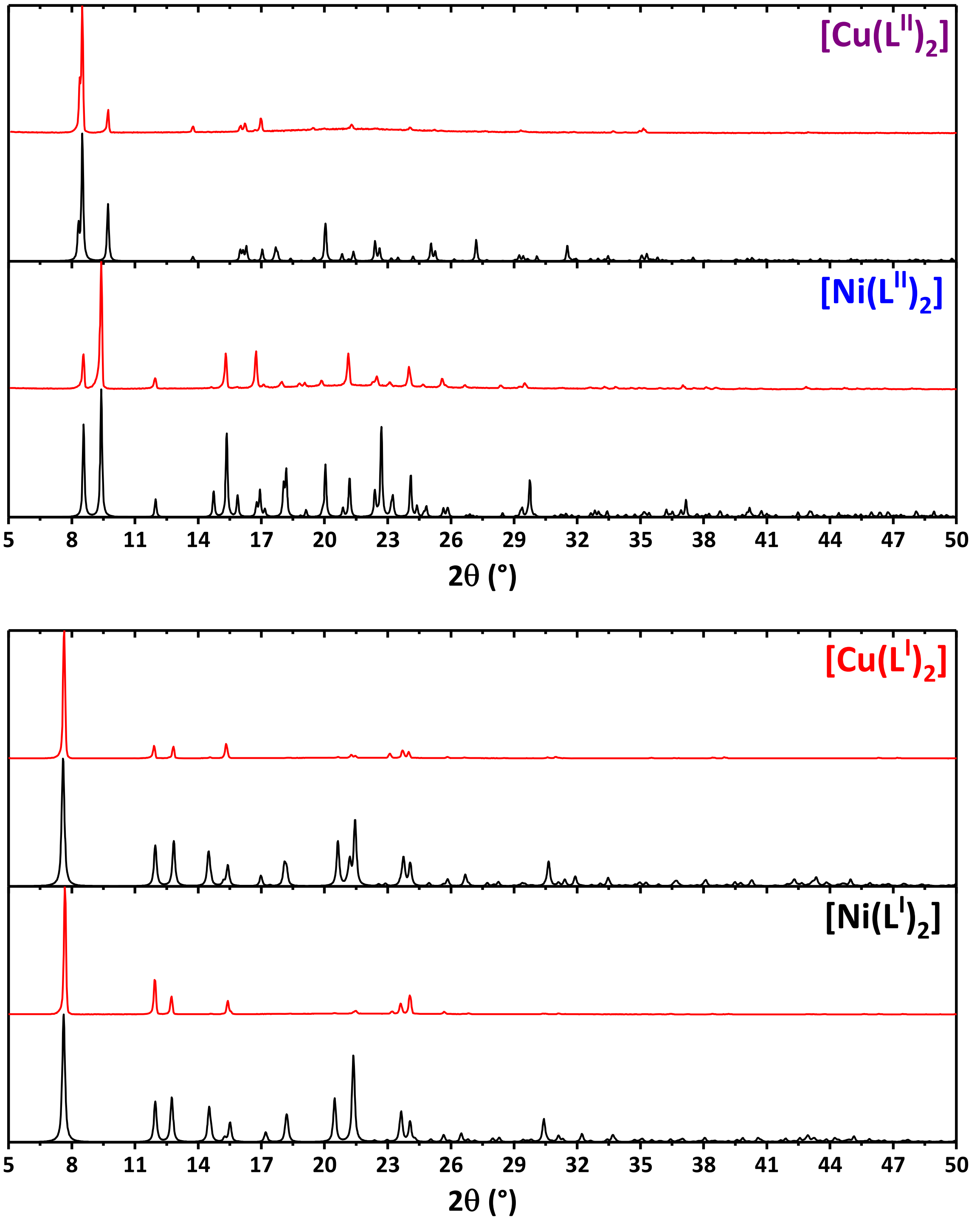

Figure S1 Calculated (black) and experimental (red) X-ray powder diffraction patterns of **[Ni(L^{I,II})$_2$]** and **[Cu(L^{I,II})$_2$]**.

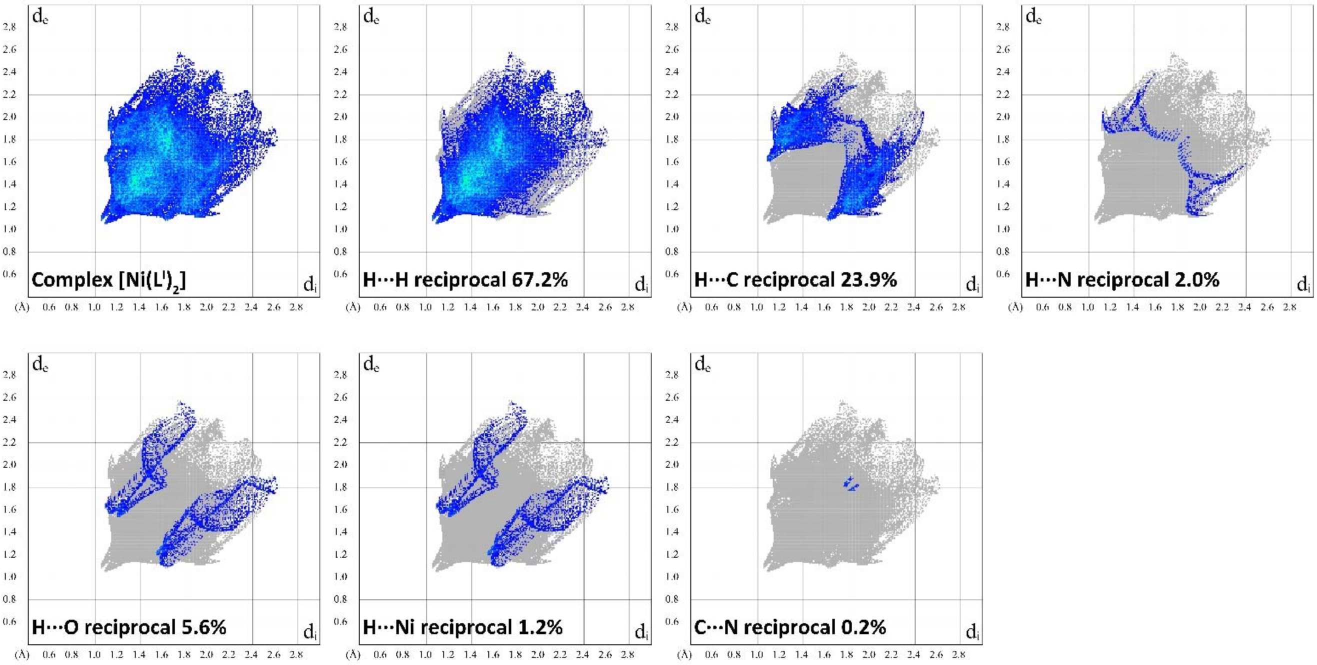

Figure S2 2D and decomposed 2D fingerprint plots of observed contacts for **[Ni(L^{I})$_2$]**.

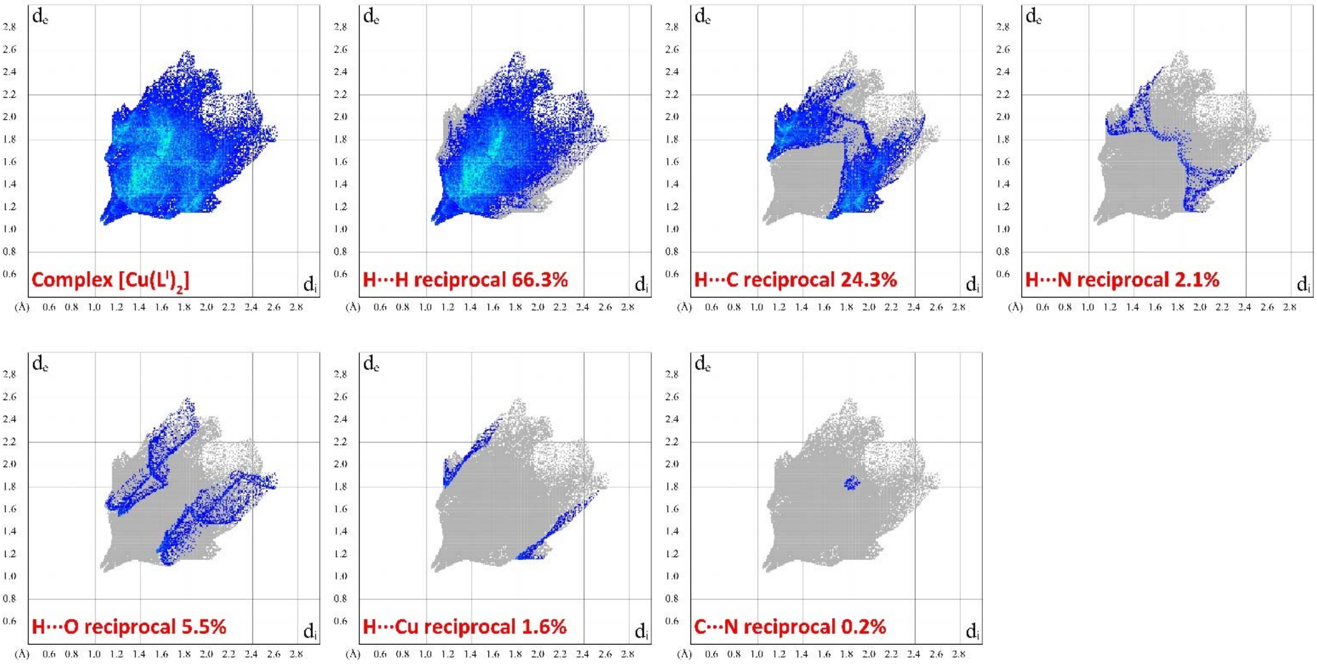

Figure S3 2D and decomposed 2D fingerprint plots of observed contacts for **[Cu(L^{I})$_2$]**.

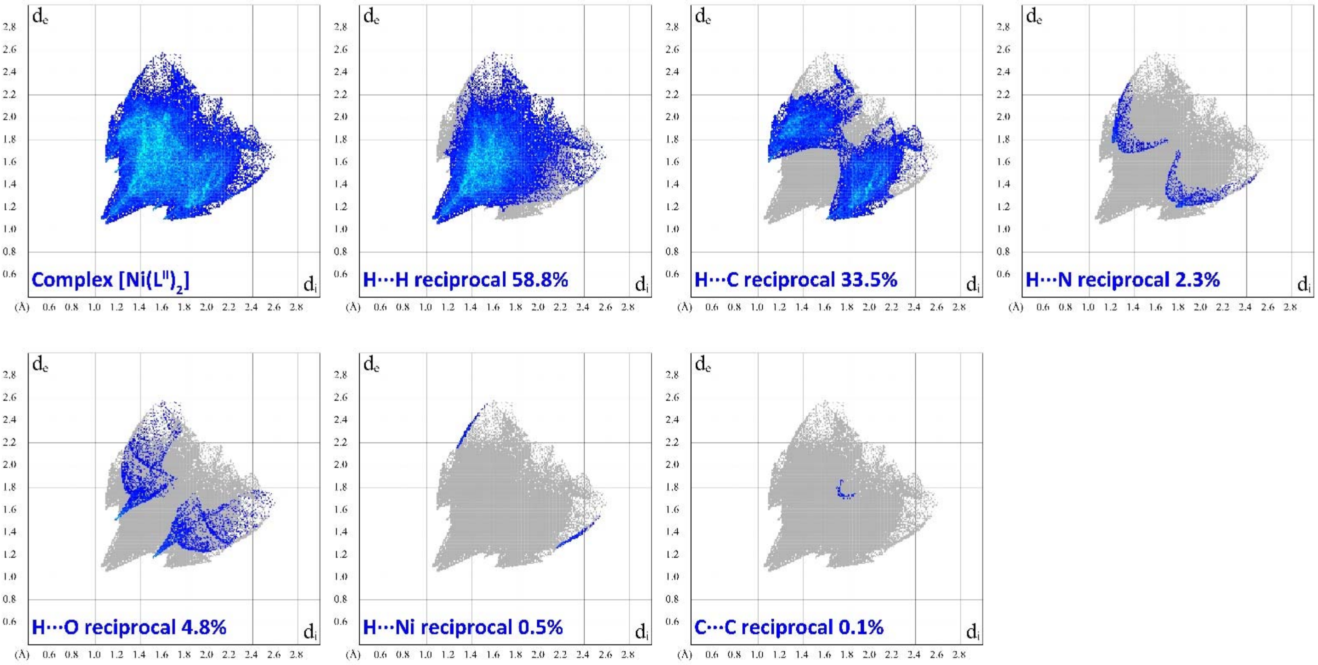

Figure S4 2D and decomposed 2D fingerprint plots of observed contacts for **[Ni(L^{II})$_2$]**.

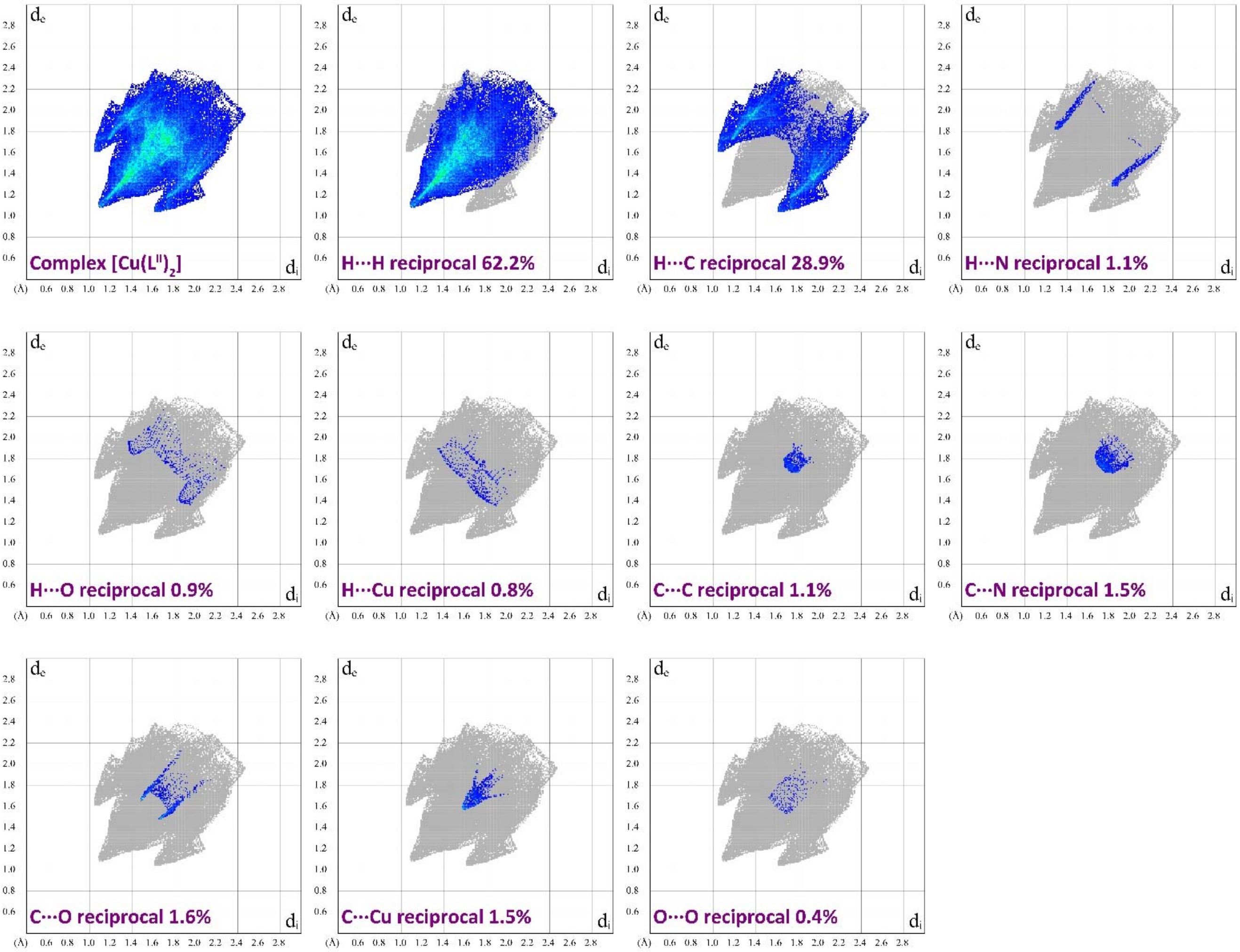

Figure S5 2D and decomposed 2D fingerprint plots of observed contacts for **[Cu(L^{II})$_2$]**.

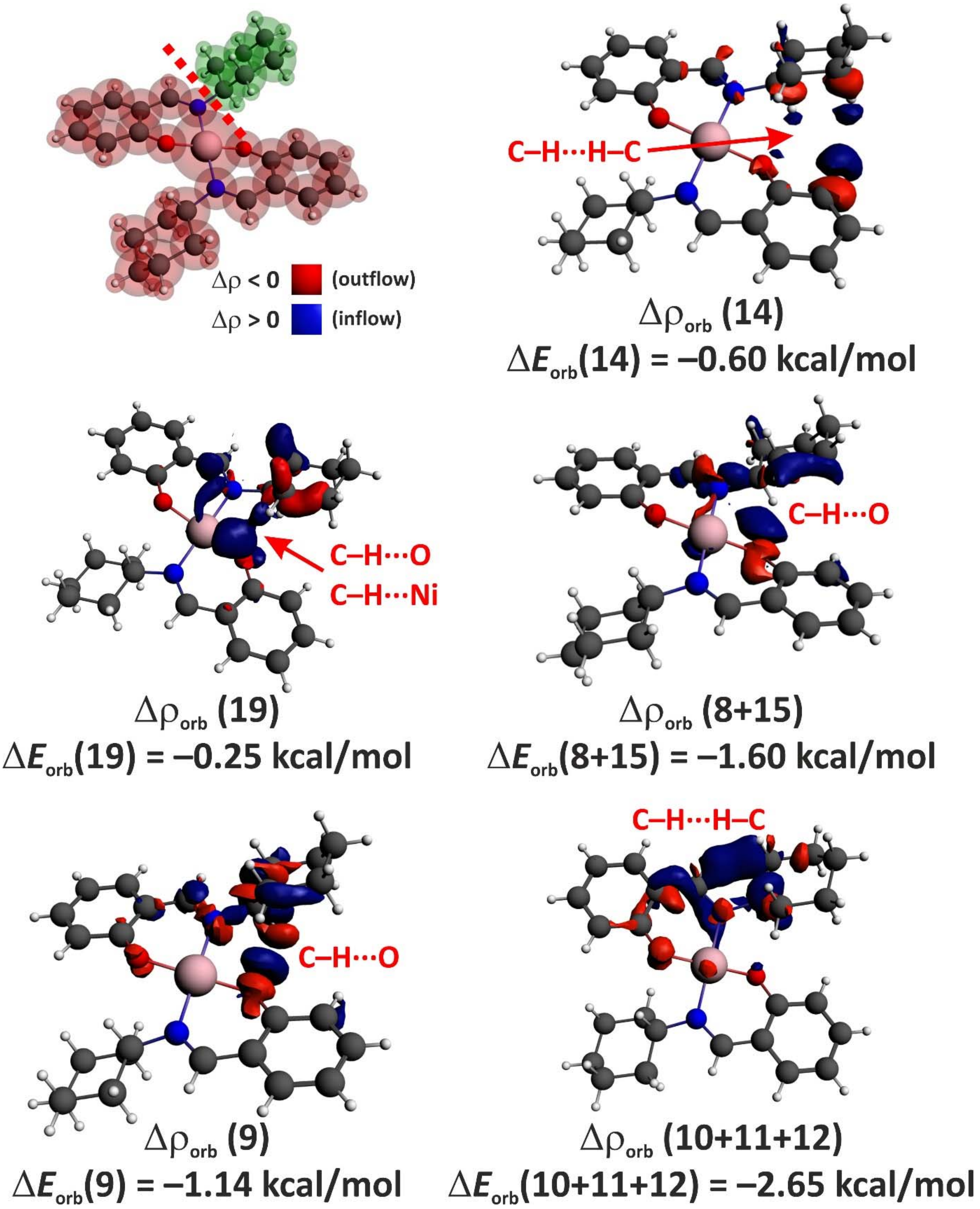

Figure S6 ETS-NOCV/BLYP-D3/TZP charge delocalization channels $\Delta\rho_{orb}$, describing intramolecular contacts together with their orbital interaction stabilizations ΔE_{orb} in the structure of **[Ni(L^{I})$_2$]**.

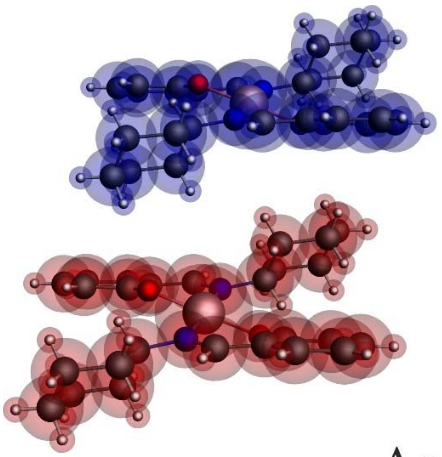

BLYP-D3(BJ)TZP	ΔE (kcal/mol)
ΔE_{Total}	–29.72
ΔE_{orb}	–14.47 (37%)
ΔE_{elstat}	–6.73 (17%)
ΔE_{Pauli}	9.35
$\Delta E_{dispersion}$	–17.87 (46%)
$\Delta E_{Total} = \Delta E_{orb} + \Delta E_{elstat} + \Delta E_{Pauli} + \Delta E_{dispersion}$	

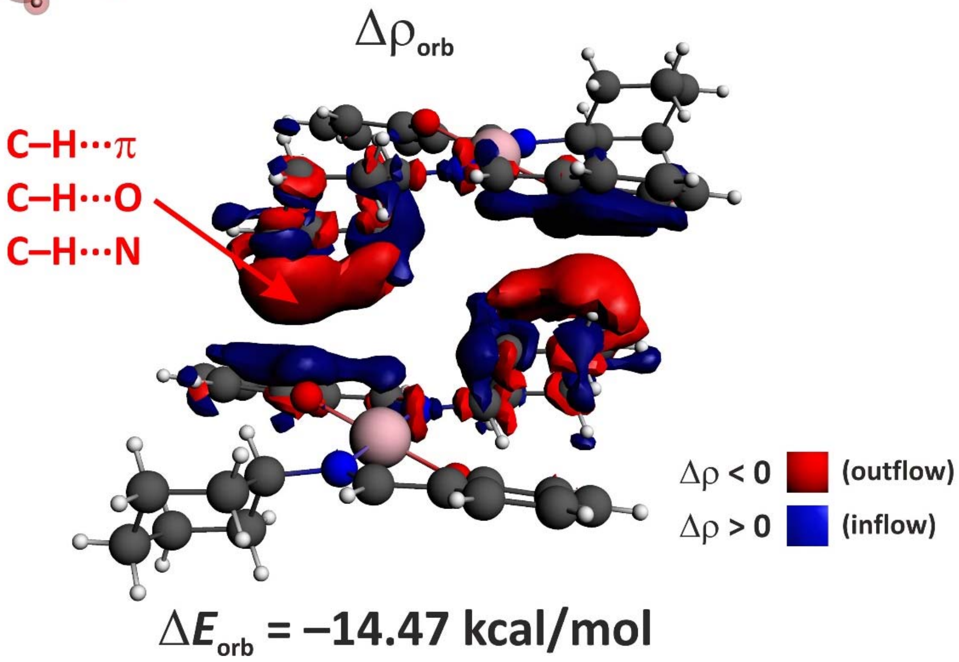

Figure S7 ETS-NOCV/BLYP-D3/TZP energy decomposition results for the crystal dimer of **[Ni(L^{I})$_2$]**. The considered model and ETS based results (top), and the overall deformation density $\Delta\rho_{orb}$ with the corresponding ΔE_{orb} (bottom).
